# Neurotrophins and their involvement in digestive cancers

**DOI:** 10.1038/s41419-019-1385-8

**Published:** 2019-02-11

**Authors:** Sabrina Blondy, Niki Christou, Valentin David, Mireille Verdier, Marie-Odile Jauberteau, Muriel Mathonnet, Aurélie Perraud

**Affiliations:** 1 Laboratoire EA3842 CAPTuR « Contrôle de l’Activation cellulaire, Progression Tumorale et Résistance thérapeutique″, Université de Limoges, Faculté de médecine, 2 Rue du docteur Marcland, 87025 Limoges, France; 2 Service de Chirurgie Digestive, Endocrinienne et Générale, CHU de Limoges, 2 Avenue Martin Luther King, 87042 Limoges, France; 3 Service de Pharmacie, CHU de Limoges, 2 Avenue Martin Luther King, 87042 Limoges, France; 4 Service d’Immunologie, CHU de Limoges, 2 Avenue Martin Luther King, 87042 Limoges, France

## Abstract

Cancers of the digestive system, including esophageal, gastric, pancreatic, hepatic, and colorectal cancers, have a high incidence and mortality worldwide. Efficient therapies have improved patient care; however, many challenges remain including late diagnosis, disease recurrence, and resistance to therapies. Mechanisms responsible for these aforementioned challenges are numerous. This review focuses on neurotrophins, including NGF, BDNF, and NT3, and their specific tyrosine kinase receptors called tropomyosin receptor kinase (Trk A, B, C, respectively), associated with sortilin and the p75 neurotrophin receptor (p75NTR), and their implication in digestive cancers. Globally, p75NTR is a frequently downregulated tumor suppressor. On the contrary, Trk and their ligands are considered oncogenic factors. New therapies which target NT and/or their receptors, or use them as diagnosis biomarkers could help us to combat digestive cancers.

## Facts

- Digestive cancers represent the most common cause of cancer deaths worldwide. Despite important progress in therapeutics, relapse and resistance still occur. It remains a research challenge to study and identify biological pathways involved in these outcomes.
- Neurotrophins BDNF, NGF, NT3, and NT4/5, and their receptors (Trk and p75NTR) are growth factors present in the nervous system. They mediate a balance between cell survival and death according to environmental conditions.
- Numerous studies have singly implicated these growth factors in digestive cancers. As far as we are aware, no study has reported the collective role of neurotrophins in the development of digestive cancers.
- According to the literature, neurotrophins and Trk receptors are considered oncogenic markers whereas p75NTR is a tumor suppressor. The use of neurotrophins as biomarkers or potentially new targets could lead to the development of new weapons for diagnosis or for improving treatments against digestive cancers.

## Open Questions

- Could neurotrophins, their receptors (Trk and p75NTR), and related-signaling pathways play a role in the development of digestive cancers?
- What type of roles do neurotrophins and their receptors play in digestive cancers?
- How could neurotrophins improve the treatment of digestive cancers?

## Neurotrophins pathway: overview

**Fig. 1:**
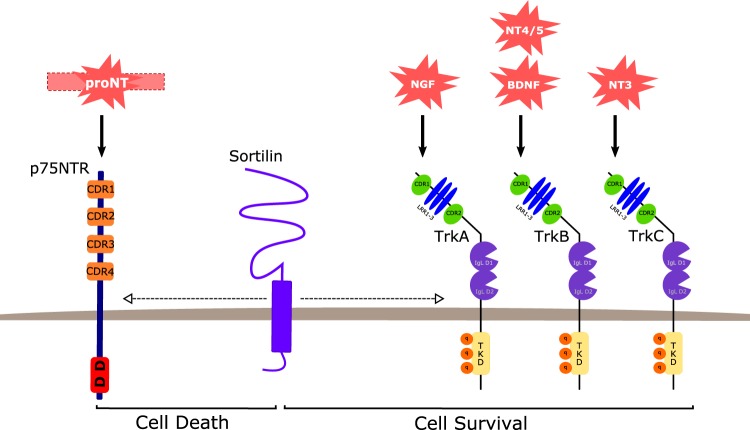
The fate of NT receptors’ family and cells following ligand binding. Trk Tropomyosin Receptor Kinase, NGF Neuronal Growth Factor, BDNF Brain-derived Neurotrophic Factor, NT Neurotrophin, CRD Cystein-Rich Domain, LRR Leucine-Rich motif, IgL Immunoglobulin-Like domain, TKD Tyrosin Kinase Domain, DD Death Domain

Signaling pathways represent current targets in cancer research for tumor diagnosis and therapy. Growth factors called neurotrophins (NT), and their tyrosine kinase receptors (tropomyosin receptor kinase (Trk)), have been described extensively in tumor development and progression. The Trk signaling pathway plays a crucial role in cancer progression and could constitute a therapeutic target for anticancer drug development^1^. Sortilin, which controls the trafficking and release of several proteins including NT and their receptors^2^, has been found overexpressed in many human cancer cells. Moreover, NT autocrine/paracrine signaling loops and sortilin/Trk cell surface interactions have been found disrupted and upregulated respectively in neurodegenerative diseases and cancers. This review focuses on NT, which has been studied for about five decades particularly in the context of pancreatic and colorectal cancers, and their suitable role as biomarkers for diagnosis and/or prognosis as well as their use as new therapeutic targets. Initially, NT were described as important regulators of neuronal survival, function, and plasticity. These neuropeptides originate from precursors, the pre-proNT (260–266 amino acids), composed of a pre-pro-domain, a pro-domain, and a mature one. The first two domains are cleaved by proteases and convertases to sequentially obtain an immature form (proNT) and then, the mature form (mNT; composed of 118–129 amino acids)^3^. Four types of NT exist: nerve growth factor (NGF), brain-derived neurotrophic factor (BDNF), neurotrophin 4/5 (NT4/5), and neurotrophin 3 (NT3). NT share common structural, chemical, and biological properties^1,3^. mNT (NGF, BDNF—NT4/5 and NT3) specifically binds to three receptor types: the Trk receptors (A, B, and C, respectively) with high affinity, p75 neurotrophin receptor (p75NTR) with low affinity, and sortilin (Fig. 1). proNT binds with strong affinity to the p75NTR/sortilin complex because sortilin recognizes a conserved motif in NT’s pro-domains. proNT and mNT may display opposite cellular functions according to their receptor binding. proNT triggers cell death whereas mNT promotes cell survival.

## Trk receptors: cell survival receptors

**Fig. 2:**
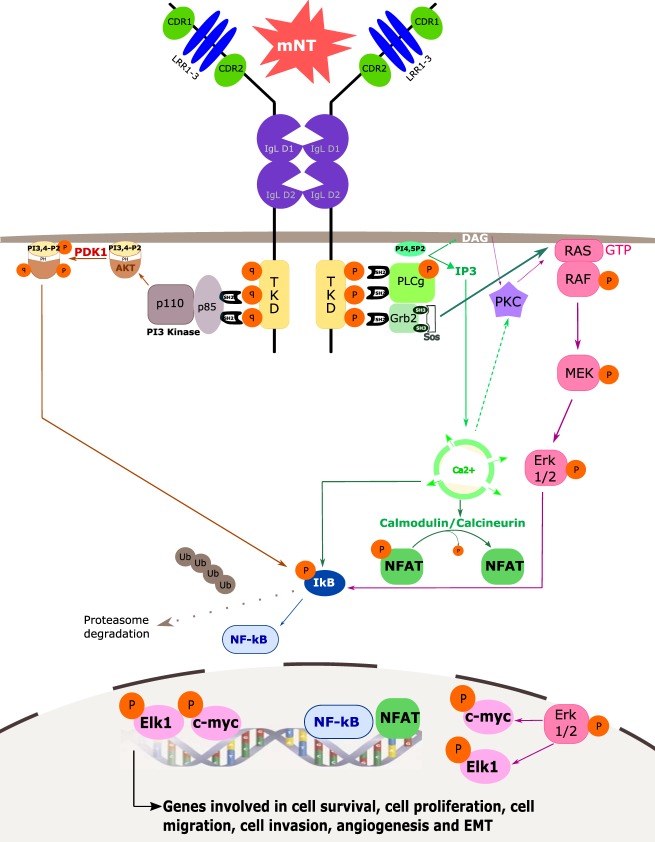
mNT/Trk pro-survival signaling involving the RAS-MAPK, PI3K/AKT, PLC gamma (γ) downstream pathways. mNT homodimers bind to their respective Trk and induce dimerization and transphosphorylation at tyrosine residues of the TKD located in the cytoplasmic tails. This phosphorylation allows the binding of sh2 domain-mediated adaptor proteins including (i) the p85-PI3K subunit, (ii) PLCγ and (iii) Grb2/SOS. (i) PI3K mediates AKT phosphorylation and activation, through PDK1. P-AKT phosphorylates IκB, triggering its ubiquitinylation and subsequent proteasome degradation, leading to NF-κB transcription factor release. (ii) PLCγ converts PI(4,5)P2 into IP3 and DAG, allowing the activation of PKC activation and, subsequently, MAPK pathways (RAF/RAF-MEK-ERK 1, 2). P-ERK 1,2 can activate IkB, which activated NF-κB, and its translocation into nucleus to activate transcription factors and/or display transcription factor functions. Simultaneously, IP3 can also trigger intracellular Ca^2+^ release, leading to the activation of NF-κB as well as NFAT, after its dephosphorylation through calmodulin/calcineurin. (iii) Finally, Grb2/SOS also activates MAPK pathways, similarly to PLCγ-PKC. All these pathways trigger transcription of genes involved in cell survival, proliferation, migration, invasion, angiogenesis, and EMT, leading to tumor growth and maintenance. Trk Tropomyosin Receptor Kinase, NT Neurotrophin, mNT mature Neurotrophin, CDR Cystein-Rich Domain, LRR Leucine-Rich motif, IgL Immunoglobulin-Like domain, TKD Tyrosin Kinase Domain, PI3K phosphatidylinositol-3-kinase, PKC Protein Kinase C, PLC Phospholipase C, PDK Phosphoinositide-Dependent Kinase, P phosphorylated residues, Ub Ubiquitin residues, DAG Diacylglycerol, IP3 Inositol Triphosphate, PI(4,5)P2 Phosphatidylinositol 4,5-biphosphate, MAPK Mitogen-Activated Protein Kinase, NFAT Nuclear Factor of Activated T-cells, EMT Epithelial-Mesenchymal Transition

Trk receptors, which are 760–810 amino acids in length, belong to the superfamily of growth factor receptors (GFR) with tyrosine kinase (TK) activities. The oncogenic role of Trk was first discovered in colorectal cancer (CRC), resulting in the discovery of TrkA, the first member of the Trk family. TrkB and TrkC were subsequently identified as a result of their homology to TrkA. The extracellular part of Trk, which consists of three leucine-rich 24-residue motifs flanked by two cysteine clusters, distinguishes them from other TK receptors. Each part of the extracellular domain of Trk is crucial for (i) NT binding and (ii) receptor dimerization upon ligand attachment. In addition to the extracellular part, Trk receptors consist of a single transmembrane (TM) domain and a cytoplasmic tail that contains TK activities like other GFR-TK. The dimerization and activation of Trk are induced by the binding of their specific ligands (mNT) or by transactivation in response to G-protein coupled receptor (GPCR) signaling as for the epidermal growth factor receptor (EGFR);^4–7^. This activation triggers several intracellular pathways involved in cell growth, survival, differentiation, and in the control of synaptic strength and plasticity of the mammalian nervous system including the mitogen-activated protein kinase (MAPK), the phosphatidylinositol-3-kinase (PI3K)/AKT, the phospholipase C gamma (PLCγ)-Ca^2+^, the nuclear factor-kappa B (NF-κB), and the protein kinase C (PKC) (Fig. 2). Alternatively, spliced or truncated forms of TrkB and TrkC without the TK domain have been described^3–5^ and behave like decoys.

## p75NTR receptor: a dual-sided receptor

**Fig. 3:**
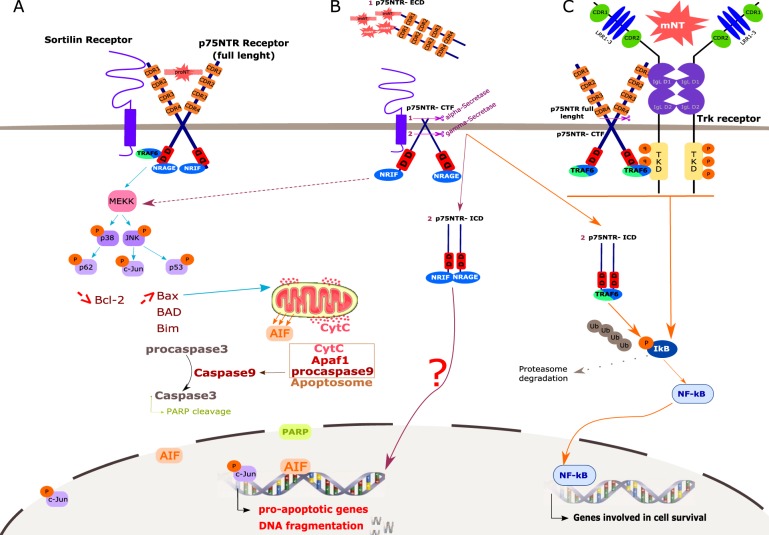
Balance between pro-apoptotic and pro-survival signaling induced by p75NTR, modulated by sortilin and the nature of binding of NT (mNT or proNT). **a** ProNT binding to the complex sortilin/ p75NTR triggers the recruitment of adaptator proteins, including TRAF6, NRAGE, and NRIF, to the p75NTR intracellular DD. These proteins activate MEKK, leading to the phosphorylation of both p38 and JNK pathways, with p62 and c-Jun, p53 activation, respectively. Altogether, these three proteins promote a decreased expression of the anti-apoptotic protein Bcl-2, concomitant with an increased expression of pro-apoptotic proteins such as Bax, Bad, and Bim. Bax translocates into the mitochondria membrane, thus forming a complex with BAK, which triggers both CytC and AIF release. Together with P-c-Jun, AIF translocate into the nucleus and induces the transcription of pro-apoptotic genes, as well as DNA fragmentation, both leading to cell death. At the same time, CytC forms the apoptosome complex with the procaspase 9 and Apaf-1, allowing caspase 9 activation (cleavage) and subsequent caspase 3 activation. Finally, the caspase 3 cleaves its substrate, the PARP, which translocates into the nucleus to exert similar effects on cell death as described above. **b** p75NTR can be cleaved by α-secretases, hence releasing its extracellular domain (p75NTR-ECD) which can bind both mNT and proNT in the extracellular media. The resting cleaved fragment anchored to cell membrane (p75NTR-CTF) and complexed to sortilin, is sufficient to induce death signaling through the same mechanisms as described above. p75NTR can also be cleaved by γ-secretase in its intracellular part, leading to the release of its intracellular domain (p75NTR-ICD) containing the DD. p75NTR-ICD can translocate into the nucleus to induce cell death but mechanisms are currently poorly understood. **c** On the contrary, after mNT binding, the p75-ICD fragment is released, as well as the CTF or full-length form of this receptor bound to the intracellular adaptator protein TRAF6 and complexed to sortilin, and can induce cell survival through the same NF-κB signaling as described previously. Trk Tropomyosin Receptor Kinase, NT Neurotrophin, mNT mature Neurotrophin, p75NTR p75 neurotrophin receptor, CDR Cystein-Rich Domain, LRR Leucine-Rich motif, IgL Immunoglobulin-Like domain, TKD Tyrosin Kinase Domain, DD Death Domain, P phosphorylated residues, Ub Ubiquitin residues, ECD Extracellular Domain, CTF C-Terminal Fragment, ICD Intracellular Domain, TRAF6 TNF Receptor associated Factor 6, NRIF Neurotrophin Receptor-Interacting Factor, NRAGE Neurotrophin Receptor-interacting MAGE homolog, MEKK MAP Kinase Kinase or MAP3K, JNK c-Jun N-terminal Kinase, Bax Bcl-2-associated X, BAD Bcl-2-associated Death promoter, Bak Bcl-2-homologous antagonist/killer, cytC: cytochrome C, PARP Poly(ADP-Ribose) Polymerase

p75NTR is a receptor, 427 amino acids in length, containing an extracellular domain, a TM domain, and a cytoplasmic tail. It belongs to the tumor necrosis factor receptor (TNFR) family due to the presence of four cysteine-rich domains in the extracellular part, which also enables conformation and ligand binding^6^. The intracellular domain contains a death domain of 80 amino acids without intrinsic catalytic activities, which are highly conserved between species. The signaling abilities of p75NTR are due to its association with cytoplasmic partners, including the neurotrophin receptor-interacting factor (NRIF) and the TNF receptor associated factor 6 (TRAF6)^6^. p75NTR can trigger two different forms of signal transduction based on ligand availability. In the presence of a ligand, p75NTR promotes either cell proliferation or differentiation, depending on ligation of mNT or proNT. However, it is noteworthy that in the absence of a ligand, p75NTR is not inactive but rather promotes active signals for cell death. Indeed, cells are sensitive to the presence of growth factors (such as NT) in the environment, and their absence leads to cell death^8,9^. The expression of p75NTR allows cells to detect the availability of growth factors in their environment. Such receptor behavior is essential in the control of tumorigenesis^6,8,9^. Signaling via p75NTR results in different biological effects depending on which adaptor proteins bind to the receptor and its ability to heterodimerize with other types of receptors (Trk, neurotensin receptors 1 and 2 (NTSR1–2) as well as sortilin)^7^. NRIF and TRAF6 recruit Rac to activate the c-Jun N-terminal Kinase (JNK) signaling cascade leading to cell death *via* further downstream events (Fig. 3a). Simultaneously, co-expression of both NRIF and TRAF6 induces translocation of NRIF from the plasma membrane to the cytosol and/or nucleus and enhances TRAF6-mediated activation of NF-κB. The latter promotes cell survival when p75NTR is co-expressed with Trk receptors (Fig. 3c)^6^. Moreover, p75NTR can undergo ectodomain shedding by α—γ secretases and TNF-α convertases (TACE) leading to the release of the p75NTR intracellular domain (p75-ICD) into the cytoplasm or nucleus. This cleavage abolishes the signaling induced by the ligand, but the carboxy-terminal tail stilled anchored to the membrane which improves Trk-mediated trophic activities^5,6^ (Fig. 3b, c).

## Sortilin: a multitasking receptor

Sortilin, also called neurotensin receptor 3 (NTSR3), is 831 amino acids long and belongs to the Vps10 protein family which includes four other members: SorLA, SorCS1, SorCS2, and SorCS3. These five proteins share a common luminal N-Terminal Vps10p domain characterized by two homologous regions of 10 cysteine residues, followed by a single TM domain and a short cytoplasmic tail which is not able to transduce signals but facilitates rapid protein internalization. Like all Vps10 protein family members, sortilin can be cleaved by PKC-dependent mechanisms, releasing the extracellular part from the plasma membrane^2,5,10,11^. Despite its predominant intracellular localization, sortilin also acts as a membrane receptor which can bind several ligands including immature and mature forms of NT^12^. Sortilin is also able to heterodimerize with several receptors such as RTK (including Trk receptors) and p75NTR as discussed above.

## Trk, p75NTR, and sortilin: partners for cell survival or death?

p75NTR and sortilin can induce both cell survival and apoptosis. p75NTR is more efficiently activated by proNT due to a higher affinity; however, it can also bind mNT. In vitro and in vivo studies in neurons have shown that upon activation by proNT, the sortilin/p75NTR complex induces apoptosis through JNK 3 and caspases 3, 9 signals and through association with several adapter proteins such as NRAGE. All these signals lead to cell cycle arrest and apoptosis^1,3^ as described above (Fig. 3a). The p75-ICD can also interact with Trk and sortilin. The juxtamembrane sequence of p75NTR binds the intracellular domain of Trk and sortilin, enhancing Trk signaling pathways with anti-apoptotic effects^6,13^ (Fig. 3c).

In summary, the binding of mNT to p75-ICD/Trk/sortilin promotes cell survival, as signaling through Trk associated with p75-ICD is stronger and can persist for longer than via Trk alone. The binding of proNT to p75NTR/sortilin leads to cell apoptosis. Indeed, the enhanced action of proNT binding to p75NTR is dependent on its association with sortilin, which binds to a conserved motif in the proNT domain^14^. On the other hand, the sortilin/p75NTR/TrkB complex plays a critical role in cell survival and particularly in cancer cell growth^10,15,16^.

Due to the diverse, numerous, and complex roles exerted by the NT and their receptors, this review presents a general overview of their functions in digestive cancers.

## Neurotrophins’ pathway in digestive cancer cells

### Esophageal and gastric cancers

Esophageal squamous cell carcinoma (ESCC) is associated with a poor prognosis as extensive local invasion or lymph node metastasis is often present at the time of diagnosis. Only 1% of patients survive beyond five years following diagnosis^17^. Similarly, gastric cancer (GC), the fourth most common malignant tumor in the world^18^, is often symptomatic only at an advanced stage. The p75NTR, NGF/TrkA (for ESCC), and BDNF/TrkB (for GC) have been extensively researched in these cancers in an effort to develop better predictive markers.

Initially, it appeared that NGF and TrkA were downregulated during tumor progression in ESCC tissues and correlated with poor differentiation and advanced tumor stages^19^. More recent studies have reported that TrkA expression is associated with lymph node metastasis, distant metastasis, higher TNM stages, poorer tumor differentiation, and poorer relapse-free survival both in ESCC and GC^20,21^. Moreover, ESCC is characterized by strong NGF expression and secretion, high expression of TrkA and its activated phosphorylated form, and negative p75NTR expression. Consequently, NGF might stimulate TrkA rather than p75NTR in aggressive cancers with strong malignant potential^20^. With regard to the BDNF/TrkB axis, a high level of TrkB expression has been observed in both cancers and is associated with tumor growth, invasiveness with distant metastases, and poor prognosis^22,23^. BDNF expression and secretion have also been observed in invasive front of primary tumors in GC patients and is strongly correlated with E-Cadherin downregulation and consequently with anoikis resistance (apoptotic cell death resulting from loss or inappropriate cell-matrix interactions^24^), invasiveness (higher cell proliferation and migration), disease progression, and poor prognosis^25,26^. In ESCC, higher levels of TrkB have also been observed in 5-FU (5-fluorouracil)- and CDDP (cisplatin)- resistant cell lines. Finally, p75NTR expression is lower, sometimes even non-existent, during esophageal carcinogenesis^27^ and in advanced and metastatic GC cells compared with normal mucosa and non-metastatic cells, which promotes cell cycle arrest^28,29^. Consequently, this receptor inhibits cancer cell metastatic capacities. Hence, when expressed, p75NTR acts as a tumor suppressor and is a good prognostic indicator. Its expression is lost with advanced cancer stages and is linked to survival and maintenance in ESCC and GC.

These data support the involvement of NGF/TrkA and BDNF/TrkB in ESCC and GC tumor growth and metastasis inducing epithelial-mesenchymal transition (EMT). TrkA and B are oncogenic factors that could be targeted as potential biomarkers for prognosis and diagnosis.

### Pancreatic cancer

Pancreatic ductal adenocarcinoma (PDAC) is the fourth most common cause of cancer-related deaths. The five-year survival rate is < 10% due to often late diagnosis, making it the most deadly of common malignancies^30^.

All NT ligands and subtypes of Trk are overexpressed in PDAC compared with healthy tissues. The NGF/TrkA axis is the most studied among neurotrophic factors in PDAC. A significant correlation has also been observed between NGF and TrkA expression and increased PDAC cell proliferation and tumorigenicity through the downstream activation of p38-MAPK signaling^31–33^. Moreover, it has been shown that TrkA is involved in PDAC chemoresistance to gemcitabine^34^. Using a nude mice metastatic model of PDAC developed by Bruns and co-workers^35^, only one study reported TrkB expression in PDAC^36^. TrkB expression would result in tumor aggressiveness, cell growth through inhibition of apoptosis, and promotion of angiogenesis favoring metastatic dissemination processes.

These data support TrkA and TrkB (at lesser extent) expression as markers of poor prognosis and tumor aggressiveness in PDAC.

### Hepatic cancer

Hepatocellular carcinoma (HCC), the main form of liver cancer, is the seventh most common cancer and the third leading cause of cancer-related death worldwide^37^. HCC has a rapid clinical course, poor response to conventional treatments, and consequently poor clinical outcomes mainly due to late diagnosis.

NT and their receptors have been poorly studied in liver cancer. These factors, especially NGF/TrkA, were first identified from liver tissue during a fibrosis injury and HCC in animal models, with contradictory results. Hepatocytes from profibrotic liver, early preneoplastic lesions, and HCC expressed NGF but not TrkA^38,39^. On the other hand, NGF and TrkA were expressed in the tissues of patients suffering from HCC (with or without cirrhosis), but not from patients with liver cirrhosis without HCC or healthy patients^40,41^. NGF is mainly localized in the cytoplasm (free, in vesicles and RE) and beneath the nuclei of hepatocytes; however, it is also found in endothelial and spindle-shaped cells. TrkA is mainly localized on the cell membrane of a few hepatocytes, and interestingly in the cytoplasm of endothelial cells and on the cell membrane of lymphocytes. Thus, NGF likely acts in both autocrine and paracrine ways as a messenger molecule in the crosstalk between different cell types in HCC^42^. One study reported the possible involvement of the three Trk as potential oncogenes in HCC^43^. In the majority of HCC patients and in metastatic HCC cell lines, the expression of Trk mRNA is significantly higher than that seen in healthy tissue or cell lines. This overexpression induced by hypomethylation of their promoters, could contribute to HCC progression with tumor cell invasion and dissemination. p75NTR has been described as a tumor suppressor in HCC as a result of the strong inverse correlation between its expression and the grade of HCC or patients stages^44^. When p75NTR is upregulated, it inhibits proliferation, growth, and tumorigenecity of HCC cells, probably through the inhibition of cell cycle progression^45^.

These data imply that NGF has a crucial role in HCC progression with TrkA functioning as an oncogene. Consequently, the use of NGF as a marker of HCC progression and also as a potential target for novel therapeutic approaches in HCC should be investigated. Moreover, these data provide further information regarding molecular mechanisms underlying the tumor suppressive functions of p75NTR, which might be used as a prognostic biomarker in HCC.

### Colorectal cancer

Colorectal cancer (CRC) is the third most commonly diagnosed cancer worldwide with 1.7 million new cases and 832,000 deaths in 2015^46^. As the majority of deaths result from late diagnosis, it is important to identify new biomarkers to allow early detection and better treatment of this disease. Recently, the consensus molecular subtypes (CMS) classification of CRC has been established based on transcriptional analysis. According to this, four major groups of CRC^47^ exist. The CMS1 group, named “MSI and Immune” (14%), displays microsatellite instability, *BRAF* hypermutation, and important immune activation. The CMS2 group, termed “Canonical” (37%), presents WNT, MYC activation pathways, and an epithelial gene expression profile (succession of loss of APC, activating mutations of *KRAS*, and loss of TP53). The CMS3 group, also known as “Metabolic” group (13%), shows high metabolic and epithelial dysregulation and *KRAS* mutations. Finally, the CMS4 group, named “Mesenchymal” (23%), is characterized by TGF-β activation, stromal invasion, and angiogenesis induction. All other CRC, that share settings from different groups, are not classified (13%). Although this new genomic signature-based classification system allows specific treatment adaptation for each group, resistance and relapse often occur.

#### Upregulation of the oncogene TrkB

TrkB is up-regulated and acts as an oncogenic factor in solid tumors (neuroblastoma, lung, prostatic, pancreatic, and ovarian cancers). It is involved in apoptosis inhibition, invasion induction, tumor proliferation via lymphangiogenesis-associated metastasis, and EMT regulation^48,49^. Consequently, the expression of TrkB is associated with poor patient outcomes. Colonic tumors express higher BDNF and TrkB transcriptomic and protein levels than non-neoplastic tissues. In addition, the activation of the BDNF/TrkB machinery in CRC cells and tissues and their critical roles in colon tumor growth have previously been demonstrated^15,50,51^. Solid tumors are starved and hypoxic due to the lack of blood vessels, which increases TrkB and sortilin expression, as well as endogenous BDNF production and release. Moreover, both BDNF/TrkB and BDNF/sortilin are co-localized to CRC cell plasma membranes. Endogenous BDNF is involved in an autocrine CRC cell survival and proliferating signaling loop involving TrkB and the AKT downstream signaling pathway. This autocrine pro-survival mechanism is counterbalanced when cells are treated with recombinant human pro-BDNF, which co-localizes with the complex p75NTR/sortilin, thus inducing and enhancing CRC cell apoptosis^15,52^. TrkB has been shown to induce EMT through Twist/Snail transcription factors^49^. Moreover, TrkB expression is inversely correlated with E-Cadherin expression and positively correlated with Vimentin, making it a potent suppressor of anoikis. Altogether, these data support the potential of TrkB in promoting CRC cell proliferation, migration, invasion, and metastasis through EMT induction^53,54^.

Patients with metastatic CRC are often treated with Cetuximab, an EGFR-targeted monoclonal antibody; however, resistance to this drug is often observed. Numerous molecular mechanisms for this have been described including the activating mutations of *KRAS* and *BRAF*^55,56^ or the amplification of RTK expression like HER2^57^. As the anti-proliferative effect of Cetuximab requires reduced BDNF/TrkB signaling, an intercommunication between both (TrkB and EGFR) pathways exists. TrkB activation could counteract the effects of Cetuximab and constitute an important resistance mechanism to this therapy^58^.

#### Downregulation of the tumor suppressor dependence receptor, TrkC

The *TrkC* promoter is methylated in 60% of adenomas, 10% of normal colon epithelium and in 100% of nine CRC cell lines, which led to the silencing of *TrkC* gene expression. Thus, NT3 expression is significantly lower in tumor samples and CRC cell lines. A strong correlation between NT3 expression and the methylation status of its promoter has also been observed, i.e., the lack of NT3 expression is linked with an aberrantly methylated *NT3* promoter. Hence, TrkC is a tumor suppressor whose expression is abrogated or largely downregulated in CRC cells. When TrkC is alone overexpressed, as for other dependence receptors, it stimulates caspase activity. The addition of NT3 and subsequent binding to TrkC results in abrogation of this caspase-dependent apoptosis and promotion of tumor growth and migration. Consequently, the tumor suppressor function of TrkC is linked to its role as a dependence receptor whose functions are linked to its KF domain^59^.

These data confirm TrkC as a conditional tumor suppressor in CRC, probably via its dependence receptor functions. These can largely be downregulated by both epigenetic (aberrant promoter methylations) and genetic (inactivating somatic mutations) mechanisms. The loss of NT3 expression precedes the loss of TrkC, allowing survival advantages for the CRC cell silenced forTrkC. Consequently, the loss of TrkC expression could occur in the initial stages of CRC and contribute to the transformation of normal epithelial cells^59^.

## Neurotrophins in the crosstalk between nerves, microenvironment, and cancer cells

Cancer cells reside in a tumor microenvironment (TME) composed of an extracellular matrix, endothelial cells, immune cells, cancer-associated fibroblasts, and cancer stem cells (CSC). Some NT are involved in CSC properties. In ESCC, p75NTR permits the identification of a small, undifferentiated cell subpopulation which expresses stem cell markers and possesses high tumorigenic potential (only 500 of p75NTR-positive cells are sufficient to form a tumor in vivo compared to 2000 p75NTR-negative cells). Only the p75NTR-positive cells are resistant to chemotherapies and are capable of self-renewal, forming spheres in vitro which contain both p75NTR-negative and -positive cells^60^.

The TME plays a crucial role in solid cancer progression. The role of nerves in this environment, particularly peripheral nerves, has been studied more recently. The network which connects the TME to the central nervous system is made up of peripheral nerves. Perineural invasion (PNI) is often linked with poor prognosis. It is the process by which (i) cancer cells invade passive nerves, disseminate and, (ii) growing nerves also infiltrate tumors enhancing cancer progression. During PNI, autonomic neurotransmitters (including NT) are released from nerves and directly impact the TME through the stimulation and activation of their specific receptors expressed by both stromal and cancer cells. Simultaneously, as a result of the expression of neurotrophic factor receptors, nerve fibers are sensitized and drawn to NT directly produced and released by cancer cells. A crosstalk between nerve and cancer cell exists and is based on the NT action. Neurotrophic factors thus display messenger functions between cancer cells and nerve fibers, and the NT/Trk signaling constitutes a driver of peritumor innervation^61^.

As recently described for GC, acetylcholine (ACh)/NGF/Trk signaling is required to promote both the growth of nerve fibers and proliferation of the gastric epithelium. The central role of NGF/Trk is in cancer initiation and progression. During early carcinogenesis, Tuft cells expand and produce ACh within the epithelium that becomes dysplastic. The ACh released from the Tuft cells promotes NGF upregulation within cancer cells expressing the ACh receptor, M3R. NGF released by cancer cells in the TME stimulates nerve fibers surrounding the niche containing the stem cells and stimulates them through the Trk receptor that they express, thus promoting an abnormal innervation from the bottom to the top of the epithelium. Later during carcinogenesis, nerve fibers produce and release ACh which contributes to the maintenance of gastrointestinal stem cells and also stimulates cancer cells through the M3R signaling. The ACh-NGF axis is a positive feedback loop which promotes the abnormal innervation observed in the TME and consequently contributes to gastrointestinal carcinogenesis, regulating both cancer cells growth and peritumoral TME remodeling^62^.

Invasion of intrapancreatic nerves occurs frequently in PDAC, promoting local and distant tumor spread. The first PDAC lesions are accompanied by a sharp increase in NGF, TrkA, BDNF, and TrkB mRNA levels within the tumors. In addition and as described above, neurotrophic factors (especially NGF) have been identified in resected PDAC, primarily from patients with advanced disease, and correlate with nerve hypertrophy and PNI^63,64^. Neurotrophic factors are known to control the germination of sympathetic neurons and primary sensory afferent fibers. Yet, pancreatic tumor cells are able to migrate through sympathetic nerves which facilitates tumor cell dissemination in PDAC and induces metastasis^65^. In accordance with these findings, NGF expression is restricted to the cytoplasm of cancer cells whereas TrkA is predominantly expressed in nerves^66^ and correlates with PNI^67^. Moreover, tumors with higher NGF expression frequently have PNI, lymph node metastasis, and hyperplasia of nerves. NGF can thus be considered as an autocrine promoter of PDAC as a result of its production by cancer cells and, a chemotaxin for nerves facilitating neural growth, contact, and high affinity with pancreatic cancer cells thus increasing PNI^67^. Depending on expression levels and the ratio of TrkA to p75NTR, NGF can have either stimulatory or inhibitory effects; this indicates communication between pancreatic cancer cells secreting NGF and surrounding nerves expressing both p75NTR and TrkA^68^. Contrary to the importance of ACh/NGF/Trk signaling in GC, a catecholamine/NGF feedforward loop is highlighted in PDAC^69^. Overexpression of TrkB in PDAC and its correlation with PNI, positive retroperitoneal margin, and liver metastatic latency in patients has only been reported in one study^36^, but nothing is known about BDNF/TrkB and NT3/TrkC involvement in PDAC-related PNI.

As for all other organs of the gastrointestinal tract, the colon is composed of autonomic extrinsic (parasympathetic, sympathetic, and sensory nerve fibers) and intrinsic (enteric nervous system) innervations. The Auerbach’s and Meissner’s plexus reinforce the ultra-innervation system of colonic wall.

The importance of severity of neural invasion (rather than its prevalence) in survival, recurrence, and progress after chemo and/or radiotherapies should be considered in CRC. In fact, lack of considerable biologic affinity between cancer cells and neurons, the low expression profile of colonic nerves for chemoattractant molecules, and the absence of major neuroplasticity in colon cancer models (cell lines and patients) may explain the lack of studies on nerve involvement in CRC^70^.

These results highlight the importance of the TME, particularly with regard to peripheral nerves and their involvement in cancer progression, and evidence the peripheral nervous system as a novel tumor treatment strategy.

## Discussion

The main challenge with regard to digestive cancers is late diagnosis, often made when the disease is already at an advanced stage. This results in non-efficient current therapies and consequently to tumor relapses. This review focuses on the different functions of NT and their receptors as oncogenic or tumor suppressor, as well as their roles in the stimulation of cancer cells via the autocrine/paracrine positive feedback loop and, in promoting PNI in the TME. Consequently, NT and their receptors constitute suitable diagnostic and/or prognostic biomarkers and also represent novel potential therapeutic targets to inhibit cancer cell growth as well as neurogenesis.

Simultaneously targeting NT signaling in PNI and cancer cells is still a suject of debate as the design of specific inhibitors of Trk, whose aberrant oncogenic expression is observed in both peripheral nerve fibers and cancer cells, is complex. Several clinical trials are thus currently underway^71^ using three pan-Trk inhibitors: the Entrectinib, the Morestinib, and the Larotrectinib^72–74^.

NT, especially NGF and BDNF, and their specific receptors (TrkA and B, respectively), display oncogenic functions in digestive cancers worldwide. Some NT, such as NGF and BDNF, are secreted in patients’ sera and plasma and thus constitute diagnostic biomarkers for hepatic cancer^39^ and CRC^53^. NGF is primarily involved in nerve growth and invasion of cancer cells by nerves, especially in GC through cholinergic signaling and in PDAC through adrenergic signaling. The blockage of NGF/Trk signaling with pan-Trk inhibitor PLX-7486 or the ablation of Tuft cells (a local source of ACh which stimulates cancer cells to produce NGF) within GC abrogates tumor development and decreases proliferation and tumorigenesis^62^. Similarly, the inhibition of NGF/TrkA signaling in PDAC with a pan-Trk inhibitor blocks NGF-dependent growth of murine PDAC cells, inhibits tumor growth, and increases the efficiency of chemotherapy. Moreover, the combination of the pan-Trk inhibitor with inhibitors of the β-adrenergic pathway (with non-selective β-blockers) greatly enhances overall survival and decreases nerve density^69^. The use of nanocluster-associated delivery of siRNA against NGF represents an innovative way to target and inhibit NGF expression specifically in PDAC, as demonstrated in mouse models where it inhibited pancreatic tumor progression^75^. The anticancer effects on PDAC growth and survival by a novel TrkA specific inhibitor, KK5101, have also recently been demonstrated^76^.

The reintroduction of pathogenetically downregulated microRNA744, which specifically targets BDNF, inhibits GC cell proliferation and invasion^77^. Indeed, a combination of both anti-EGFR and anti-TrkB together with currently available chemotherapies, could represent novel therapeutic strategies to increase sensitivity to anti-EGFR therapies. In addition, acting on their ligands would be very promising. These factors are very often secreted by cancer cells and act in both autocrine and paracrine pathways by promoting survival, proliferation, migration, and dissemination with EMT induction.

On the contrary to Trk receptors, p75NTR acts as a tumor suppressor and promotes good prognosis. The expression of p75NTR is often very reduced in the advanced stages of cancer leading to cell survival and maintenance of cancer aggressiveness. Further research is required to determine (i) why the expression of p75NTR is lost during cancer progression (hypermethylation status) and (ii) if by specifically targeting p75NTR, it is possible to reactivate it and consequently reduce and stop cancer progression. p75NTR might also constitute a good CSC and prognostic biomarker in ESCC as its expression is restricted to these cells and is associated with a poor survival index and recurrences. Thus, specifically targeting the p75NTR-positive cancer cells in combination with systemic chemotherapies, might be a promising therapeutic strategy.

The roles of the neuropeptides TrkC, NT3, and sortilin in cancer progression are more complex. TrkC, the least studied of the Trk receptors, is a dependence receptor with oncogenic and tumor suppressing functions. When TrkC is expressed alone, without its ligand (NT3 expression was lower in tumor samples than in non-neoplasic tissues, due to its promoter hypermethylation), it induces pro-apoptotic signaling. However, TrkC expression is often downregulated (similarly to p75NTR) in CRC, abrogating its pro-apoptotic and tumor suppressor functions. This downregulation is also explained by the promoter hypermethylation status. These results reinforce the potential to use methylation inhibitors^78^ to reactivate tumor suppressing properties (as forTrkC), and reestablish the pro-apoptotic effect on cancer cells. When TrkC binds to NT3 however, it exhibits anti-apoptotic functions and acts as an oncogenic factor. Further studies are needed to better understand the role of TrkC in other cancers, particularly as it has only been well described in CRC. Sortilin, the most complex receptor of all, has not been extensively studied in cancers. It is expressed ubiquitously in cells and plays a very important role in the intracellular (endocytosis, sorting) and extracellular (exocytosis, secretion) trafficking of proteins (e.g., receptors, ligands) and also in cell signaling events such as heterodimerization with Trk receptors (e.g., TrkB to enhance the pro-survival effects of BDNF in CRC).

To conclude, NT and their Trk receptors are often oncogenic in digestive cancers and promote aggressiveness and tumor development. NT also represent key messenger molecules in the crosstalk between cancer cells and peripheral nerve fibers strongly involved in PNI; the latter is a poor prognostic indicator. On the contrary, p75NTR is the only tumor suppressor factor in all digestive cancers (Table 1). Thus, NT and their Trk receptors constitute attractive new therapeutic targets. Moreover, secreted ligands are very promising as biomarkers and should inform further research with regard to digestive cancers.

**Table 1:** Overview of the implication of NT and their receptors in digestive cancers

Neurotropins and receptors	Disease	Function (oncogenic or tumor supressor) and expression degree	References
NGF	Esophageal cancer	Unfavorable prognosis factor, highly expressed	^20^
	Pancreatic cancer	Unfavorable prognosis factor, highly expressed, promoting PNI	^66– 68^
	Hepatic cancer	Unfavorable prognosis factor, highly expressed and screted in sera	^38, 39^
TrkA	Esophageal cancer	Oncogenic factor, highly expressed	^20^
	Gastric cancer		^21^
	Pancreatic cancer		^31– 33, 63^
	Hepatic cancer		^40– 43^
BDNF	Gastric cancer	Unfavorable prognosis factor highly expressed and secreted	^25, 26^
	Colorectal cancer		^15, 50, 51, 53^
TrkB	Esophageal cancer	Oncogenic factor, highly expressed	^23^
	Gastric cancer		^21, 22, 25^
	Pancreatic cancer		^36, 63^
	Hepatic cancer		^43^
	Colorectal cancer		^15, 50, 51, 53, 54, 58^
NT3	Colorectal cancer	Weak expression	^59^
TrkC	Gastric cancer	Oncogenic factor	^21^
	Pancreatic cancer		^63^
	Hepatic cancer		^43^
	Colorectal cancer	Tumor suppressor and dependence receptor	^59, 79^
p75^NTR^	Esophageal cancer	Tumor suppressor weakly expressed	^27^
	Gastric cancer		^28, 29^
	Pancreatic cancer		^68^
	Hepatic cancer		^44, 45^
	Colorectal cancer		^15^
Sortilin	Colorectal cancer	—	^10^
